# Critical functions for STAT5 tetramers in the maturation and survival of natural killer cells

**DOI:** 10.1038/s41467-017-01477-5

**Published:** 2017-11-06

**Authors:** Jian-Xin Lin, Ning Du, Peng Li, Majid Kazemian, Tesfay Gebregiorgis, Rosanne Spolski, Warren J. Leonard

**Affiliations:** 1 Laboratory of Molecular Immunology and the Immunology Center, National Heart, Lung, and Blood Institute, National Institutes of Health, Bethesda, MD 20892-1674 USA; 2 Present Address: Department of Biochemistry and Computer Science, Purdue University, West Lafayette, IN 47906 USA

**Keywords:** Cytokines, NK cells, Immunosurveillance, Transcription

## Abstract

Interleukin-15 (IL-15) is essential for the development and maintenance of natural killer (NK) cells. IL-15 activates STAT5 proteins, which can form dimers or tetramers. We previously found that NK cell numbers are decreased in *Stat5a*−*Stat5b* tetramer-deficient double knockin (DKI) mice, but the mechanism was not investigated. Here we show that STAT5 dimers are sufficient for NK cell development, whereas STAT5 tetramers mediate NK cell maturation and the expression of maturation-associated genes. Unlike the defective proliferation of *Stat5* DKI CD8^+^ T cells, *Stat5* DKI NK cells have normal proliferation to IL-15 but are susceptible to death upon cytokine withdrawal, with lower *Bcl2* and increased active caspases. These findings underscore the importance of STAT5 tetramers in maintaining NK cell homoeostasis. Moreover, defective STAT5 tetramer formation could represent a cause of NK cell immunodeficiency, and interrupting STAT5 tetramer formation might serve to control NK leukaemia.

## Abstract (web summary)

IL-15 signals through STAT5 to modulate natural killer (NK) cell maturation, function and homeostasis, but the specific contributions of STAT5 dimers and tetramers are still unclear. Here the authors show that, while STAT5 dimers are sufficient for NK development, STAT5 tetramers are essential for NK homoeostasis by modulating apoptosis.

## Introduction

STAT5A and STAT5B are signal transducers and activators of transcription (STAT) family proteins^1,2^. These transcription factors are critical for the actions of many cytokines, including growth hormone, prolactin, erythropoietin, haematopoietic cytokines (such as IL-3, IL-5 and GM-CSF) and immune cytokines (such as IL-2, IL-7, IL-9, IL-15 and TSLP)^3^. The formation of STAT5 dimers depends on bivalent interactions between a key C-terminal phosphotyrosine of each STAT5 monomer and the SH2-domain of the other monomer, allowing the STAT5 dimer binding to γ-interferon activated sequence (GAS) motifs^1,2^. Additionally, STAT5 proteins^4,5^, analogous to STAT1 and STAT4^6–8^, can form tetramers by an N-terminal region (N-domain)-mediated interaction between two dimers, which allows binding to lower affinity tandemly linked non-consensus GAS motifs.

We have previously shown that mutant STAT5 proteins that cannot form tetramers are expressed at a similar level to WT STAT5 proteins and can be phosphorylated in response to IL-2 stimulation^9^. To determine the importance of STAT5 tetramerization in vivo, we also identified and mutated residues in the STAT5A and STAT5B N-domains that are critical for tetramerization and generated *Stat5a* and *Stat5b* single knockin and *Stat5a/Stat5b* double knockin (DKI) mice^9^. In marked contrast to the perinatal lethality observed in *Stat5a*/*Stat5b* double knockout mice^10^, STAT5 tetramer-deficient DKI mice survive and develop normally^9^. However, these mice have fewer CD8^+^ T cells, and have defective CD8^+^ T-cell proliferation in vitro, as well as in response to acute infection with lymphocytic choriomeningitis virus (LCMV) in vivo^9^. CD4^+^CD25^+^ cells were also diminished in number in *Stat5* DKI mice, with attenuated regulatory T (Treg) cell function in a model of inflammatory bowel disease^9^. In addition to these T cell defects, we also observed decreased numbers of splenic natural killer (NK) cells, but the basis for this defect and the functional activity of *Stat5* DKI NK cells was not explored.

NK cells are vital to innate immunity through their cytolytic activity and ability to eliminate tumour cells and pathogen-infected cells^11–15^, and also contribute to adaptive immune responses, particularly through their production of pro-inflammatory (TNF and IFNγ) and immunosuppressive (IL-10) cytokines as well as chemokines^13,16^. Conventional NK cells develop and mature in the bone marrow, where IL-15 promotes their differentiation, maturation, survival and expansion^11,17^. IL-15 binds with high affinity to the IL-15 receptor α chain (IL-15Rα)^18^ and signals primarily via its trans-presentation^19,20^ by IL-15Rα to a heterodimer consisting of the IL-2 receptor β chain (IL-2Rβ) and common cytokine receptor γ chain (γ_c_)^21,22^, although *cis* signalling can also occur when all three receptor chains are co-expressed^23^. The essential functions of IL-15 signalling in the development, maturation, survival and expansion of NK cells are underscored by the findings that deletion of either *Il15* ^24^ or *Il15ra* ^25^ causes profoundly defective NK-cell development. Interestingly, mice lacking *Il15* ^24^ have fewer NK cells than mice lacking *Il15ra* ^25^, consistent with the ability of IL-15 to signal via either IL-2Rβ/γ_c_ dimeric or IL-15Rα/IL-2Rβ/γ_c_ trimeric receptor complexes on NK cells.

There are fewer NK cells in mice lacking either *Stat5a* or particularly *Stat5b* ^26^, and mice lacking both *Stat5a* and *Stat5b* are essentially devoid of NK cells^10^. Because NK cells develop in *Stat5* DKI mice, albeit in decreased numbers, we could use these animals to investigate the biological actions of STAT5 tetramers and dimers in NK cell development and function. Whereas STAT5 dimers are sufficient for the early development of conventional NK cells and cytotoxicity, STAT5 tetramers are required for the later stages of maturation of conventional NK cells in bone marrow and spleen, and for the development of thymic NK cells. Interestingly, STAT5 tetramers are not required for NK cell expansion but are required for maintaining expression of anti-apoptotic proteins and suppression of pro-apoptotic proteins, and thus for NK cell survival. The decreased expression of BCL2 in *Stat5* DKI NK cells is associated with increased levels of active caspases that initiate NK cell death. Our data thus reveal that both a partial block of NK maturation and increased NK cell death contribute to the lower NK cell numbers observed in *Stat5* DKI mice, underscoring the critical functions of STAT5 tetramers in the maturation and survival of NK cells.

## Results

### STAT5 tetramers are required for normal NK cell numbers

**Fig. 1:**
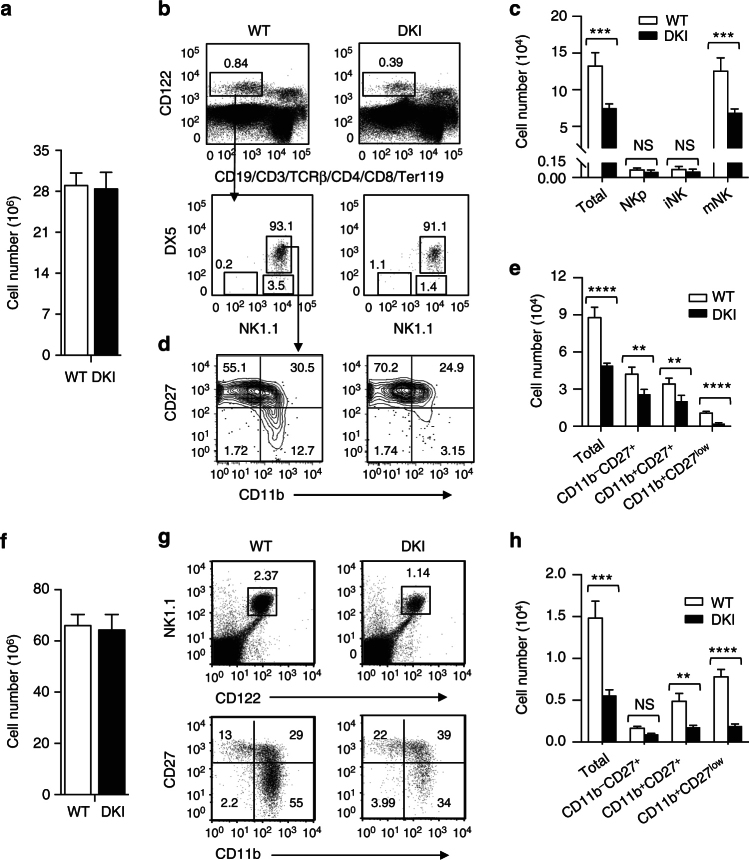
Decreased bone marrow and splenic NK cells in *Stat5* double knockin (DKI) mice. **a** Total bone marrow cell numbers from 8 WT (open bar) and 8 *Stat5* DKI (filled bar) mice after removal of red blood cells. **b** Representative flow cytometric data of total bone marrow NK cells (lin(CD19, CD3, TCRβ, CD4, CD8, Ter119)^−^CD122^+^) (upper panels) and progenitor (NK1.1^−^DX5^−^), immature (NK1.1^+^DX5^−^), and mature (NK1.1^+^DX5^+^) NK cells (lower panels) in WT and *Stat5* DKI (DKI) bone marrow. The numbers are the percentage of gated populations. **c** Total bone marrow NK (Total), NK progenitor (NKp), immature NK (iNK) and mature NK (mNK) cell numbers from 8 WT (open bars) or 8 DKI (filled bars) mice. **d** Representative flow cytometric data of total bone marrow NK cells from WT and *Stat5* DKI mice gated as lin^-^CD122^+^NK1.1^+^DX5^+^ (lower panels of **b**) and further characterised based on CD11b and CD27 staining. **e** Numbers of total bone marrow NK cells (Total) and CD11b^−^CD27^+^, CD11b^+^CD27^+^, and CD11^+^CD27^low^ populations from 8 WT (open bars) and 8 *Stat5* DKI (filled bars) mice. **f** Total splenocyte numbers from 8 WT (open bar) and 8 *Stat5* DKI (filled bar) mice, after removing red blood cells. **g** Representative flow cytometric data of total splenic NK cells from WT and *Stat5* DKI mice gated as CD3^−^CD122^+^NK1.1^+^ (upper panels) and further characterised based on CD11b and CD27 staining (lower panels). **h** Number of total splenic NK cells (Total), CD11b^−^CD27^+^, CD11b^+^CD27^+^, and CD11^+^CD27^low^ populations from 8 WT (open bars) and 8 DKI (filled bars) mice. Error bars in **c**, **e** and **h** are means ± SEM and statistical analyses were performed by grouped multiple *t*-test using Prism 7.0b

To study the function of STAT5 tetramers in the development of conventional natural killer (NK) cells, we initially compared the maturation status of NK cells in bone marrow and spleen in WT and *Stat5* DKI mice. The total numbers of WT and *Stat5* DKI bone marrow cells were similar (Fig. 1a); however, in the *Stat5* DKI mice, there was a ~50% decrease in the frequency of bone marrow lin^−^CD122^+^ total NK cells (Fig. 1b, upper right versus upper left panel), without a significant change in the frequency of NK1.1^+^DX5^+^ mature NK cells (Fig. 1b, lower panels), resulting in fewer total *Stat5* DKI NK cells (Fig. 1c). The lin^−^CD122^+^NK1.1^+^DX5^+^ mature NK cells (mNK) were substantially decreased, whereas lin^−^CD122^+^NK1.1^−^DX5^−^progenitor NK (NKp) and lin^−^CD122^+^NK1.1^+^DX5^−^immature NK (iNK) cells were not significantly affected (Fig. 1c).

Based on cell surface expression of CD11b and CD27, mouse conventional NK cells can be further divided into four maturation subsets, from CD11b^−^CD27^−^NK cells, through CD11b^−^CD27^+^ and CD11b^+^CD27^+^ NK cells, to terminally differentiated CD11b^+^CD27^low^ mature NK cells^27–29^. In *Stat5* DKI bone marrow, the frequency of terminally differentiatedCD11b^+^CD27^low^ mature NK cells was markedly decreased, whereas CD11b^−^CD27^+^ NK cells were increased (Fig. 1d), underscoring the importance of STAT5 tetramers for NK cell maturation beyond the CD11^−^CD27^+^ stage. In fact, the number of each subpopulation of NK cells was decreased, but the defect was greatest in the most mature *Stat5* DKI NK cells (Fig. 1e). Analogous to bone marrow, total splenocyte numbers were similar in WT and *Stat5* DKI mice (Fig. 1f), but total NK cell frequency (Fig. 1g, upper panels) and numbers (Fig. 1h; ref. ^9^) were lower in *Stat5* DKI NK mice, with a partial block in maturation (Fig. 1g, lower panels), with the greatest defect in terminally differentiated CD11b^+^CD27^low^ NK cells (Fig. 1h).

Because IL-7 activates STAT5 proteins^30^ and thymus-derived NK cell homoeostasis depends on IL-7^31^, we also examined thymic NK cells. The frequency of total (lin^−^CD122^+^) thymic NK cells was moderately decreased in *Stat5* DKI mice **(**Supplemantary Fig. 1a, upper panels), whereas the frequency of mature (CD122^+^NK1.1^+^DX5^+^) thymic NK cells showed little if any decrease (Supplementary Fig. 1a, lower panels). Nevertheless, the number of these cells was significantly lower in *Stat5* DKI than in WT mice (Grouped Multiple *t*-test, *p* < 0.001, Supplementary Fig. 1b **)**. IL-7Rα expression was normal (Supplementary Fig. 1c, d), so we attribute the decrease in thymic NK cell numbers to defective STAT5 tetramer formation. Thus, STAT5 tetramers are essential for the maintenance and/or expansion of thymic as well as bone marrow and splenic NK cells.

### STAT5 tetramers mediate NK cell-related gene expression

**Fig. 2:**
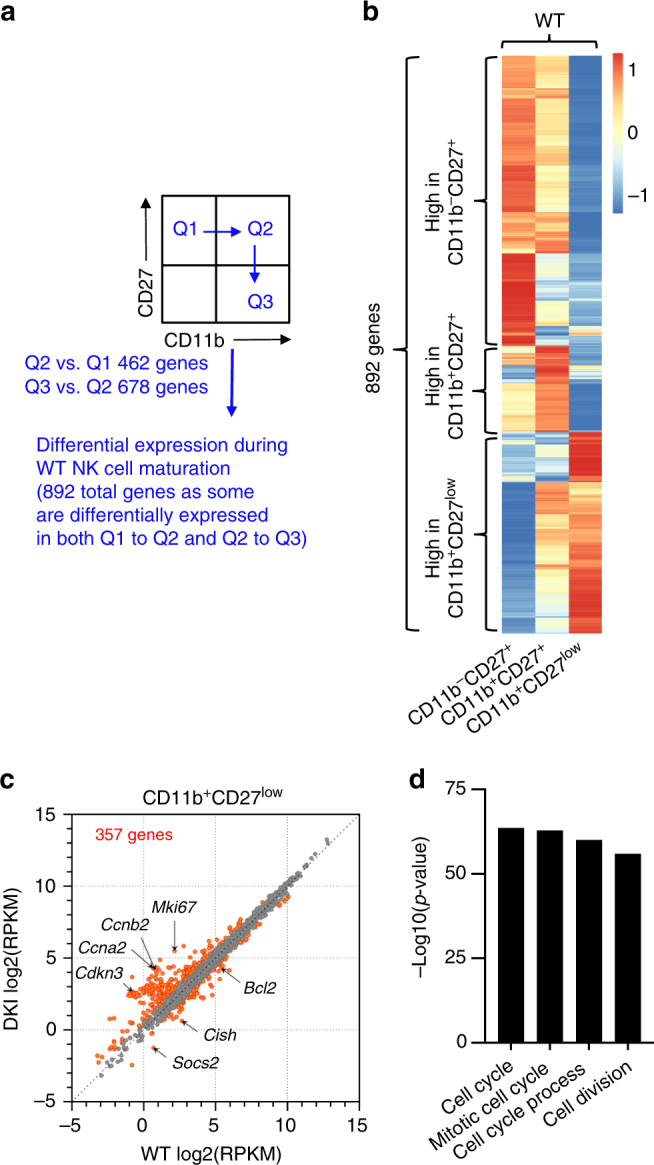
Altered gene expression in *Stat5* double knockin (DKI) NK cells. **a** Schematic illustration of NK maturation; we identified genes differentially regulated between Q1 to Q2 and Q2 to Q3 in WT NK cells, where Q1 = CD11b^−^CD27^+^, Q2 = CD11b^+^ CD27^+^, and Q3 = CD11b^+^CD27^low^. **b** Heatmap showing differentially expressed genes in WT NK subpopulations, as illustrated in **a**. Genes expressed higher in each WT subpopulation were indicated by black curly brackets on the left. The heatmap colours in each row are proportional to RPKM values. **c** Scatter plot showing all genes in grey and differentially expressed genes in red between WT and *Stat5* DKI CD11b^−^CD27^low^ NK cells. **d** Bar graph showing the 4 gene sets most enriched in the gene list of **c** based on the Gene Set Enrichment Analysis algorithm (GSEA, Broad Institute, Boston, MA)

Because mature CD11b^+^CD27^low^ NK cells were significantly reduced in *Stat5* DKI bone marrow and spleen, we next performed RNA-Seq using sorted splenic NK cells to identify the genes whose expression correlated with the CD11b^−^CD27^+^ to CD11b^+^CD27^+^ (Q1−Q2) and CD11b^+^CD27^+^ to CD11b^+^CD27^low^ (Q2 to Q3) transitions (as illustrated in Fig. 2a). First, we compared expression profiles in these populations of WT NK cells (purity of sorted NK populations ranged from 92 to 100%, Supplementary Fig. 2a, b) and identified a total of 892 genes that were differentially expressed during these transitions (Reads Per Kilobase of transcript per Million mapped reads (RPKM) ≥5 in at least one population and fold change (FC) ≥1.5)(Fig. 2a, b, Supplementary Data 1a). Of these, 462 genes were differentially expressed during the CD11b^−^CD27^+^ to CD11b^+^CD27^+^ transition and 678 genes during the CD11b^+^CD27^+^ to CD11b^+^CD27^low^ transition, with some (248 genes) differentially expressed in both transitions (Fig. 2a, Supplementary Data 1b, c). These included 82 genes encoding transcription factors (TFs) (Supplementary Fig. 2c, Supplementary Data 1f) such as *Tox*, *Klf4*, and *Prdm1* (Supplementary Fig. 2c, d) and 77 genes encoding cytokines, chemokines, their receptors, and NK receptors such as inhibitory NK receptor *Klrg1*, activating NK receptor *Cd160*, and *Il7r* (Supplementary Fig. 2e, f, Supplementary Data 1g).

Interestingly, a number of genes encoding transcription factors (Supplementary Fig. 2c) and cytokines/chemokines/receptors/NK receptors (Supplementary Fig. 2e) had altered expression in *Stat5* DKI NK subpopulations as compared to cells from WT mice (Supplementary Fig. 3a−f, respectively); whether they are responsible for the maturation defects seen in *Stat5* DKI NK cells remains to be elucidated.

Most of the 892 genes that were differentially expressed during the CD11b^−^CD27^+^ to CD11b^+^CD27^+^ and/or CD11b^+^CD27^+^ to CD11b^+^CD27^low^ transitions had similar expression patterns in corresponding WT and *Stat5* DKI NK populations, but some genes, including those encoding a number of cytokine signalling molecules (e.g., *Cish* and *Socs2*) and anti-apoptotic protein (*Bcl2*) were expressed at lower levels in *Stat5* DKI NK cells (Fig. 2c). Interestingly, a number of genes, including some of those involved in cell cycle progression, were downregulated during the CD11b^+^CD27^+^ to CD11b^+^CD27^low^ transition in WT but not in *Stat5* DKI NK cells. For example, *Mki67*, *Ccna2*, *Ccnb2* and *Cdkn3* were still expressed at higher levels in *Stat5* DKI CD11b^+^CD27^low^ NK cells than those in corresponding WT NK cells (Fig. 2c and Supplementary Data 1d). A Gene Set Enrichment Analysis of the 357 differentially expressed genes in WT versus *Stat5* DKI CD11^+^CD27^low^ NK cells revealed that the top 4 enriched gene sets are involved in cell cycle progression (Fig. 2d). Surprisingly, most of the genes (e.g., *Il2rb*, *Il2rg*, *Il15ra*, *Il7r*, *Stat5a*, S*tat5b*, *Jak*3, *Eomes*, *Elf4, Ets1, Ets2, Nfil3, Gata3*, *Id2, Irf1*, *Irf2*, *Klf4*, *Prdm1*, *Tbx21* and *Tox*)(Supplementary Fig. 4a) known to be critical for NK development and/or maturation^24,26,32–44^ were similarly expressed in CD11b^−^CD27^+^ (Supplementary Fig. 4b), CD11b^+^CD27^+^ (Supplementary Fig. 4c), and CD11b^+^CD27^low^ (Supplementary Fig. 4d) subpopulations of WT and *Stat5* DKI NK cells (Supplementary Data 1e). Expression of the genes encoding JAK1, JAK2 and JAK3 and all seven STAT proteins was similar in CD11b^−^CD27^+^ (Supplementary Fig. 4e), CD11b^+^CD27^+^ (Supplementary Fig. 4f), and CD11b^+^CD27^low^ (Supplementary Fig. 4g) WT and *Stat5* DKI NK cells (Supplementary Data 1e).

### STAT5 tetramers are needed for cytokine-mediated NK survival

**Fig. 3:**
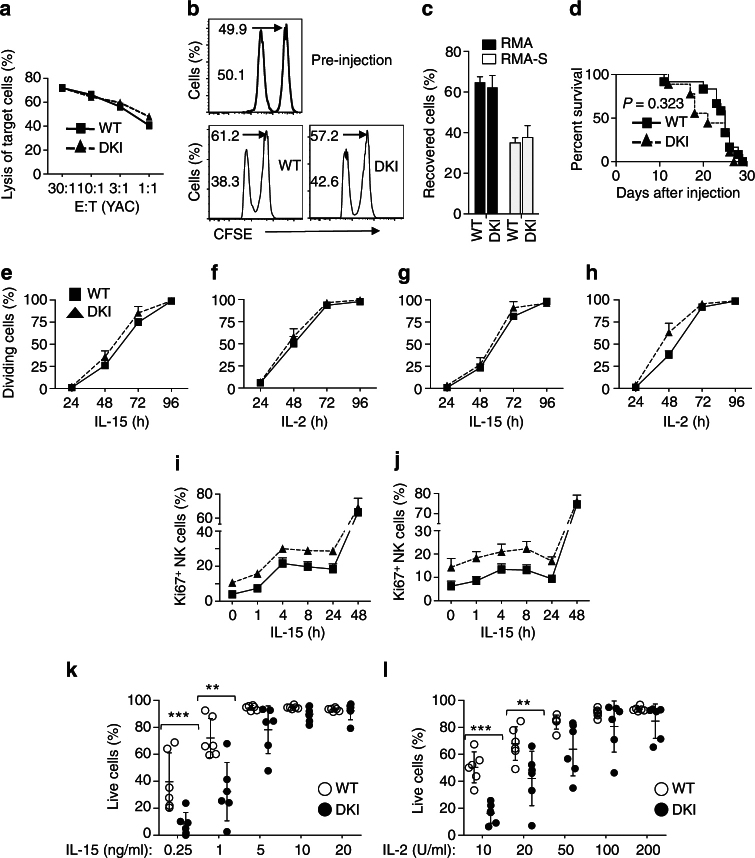
*Stat5* DKI NK cells have normal NK cytotoxicity and proliferative responses but defective survival in response to IL-2 and IL-15 stimulation. **a** Representative line graph showing the percentage of ^51^Cr-labelled YAC-1 cell lysis by WT (black squares and black lines) and *Stat5* DKI (black triangles and dashed lines) NK cells. NK cells were isolated from three WT and *Stat5* DKI mice. The experiment was performed twice. **b** Representative histograms showing percentage of CFSE-labelled RMA-S (first (left) peaks) and CFSE-labelled RMA (second (right) peaks) cells pre-injection or 16 h after intraperitoneal injection into WT and *Stat5* DKI mice. The numbers indicate the percentage of RMA-S and RMA cells recovered. **c** Summary of two independent experiments. **d** Kaplan−Meier curves showing similar RMA-S tumour rejection in WT (*n* = 12, solid squares with line) and *Stat5* DKI (*n* = 9, solid triangles with dotted line) mice. The data are derived from three independent experiments and the *p*-value (Mantel-Cox test) is shown. Black squares and solid lines indicate WT cells and black triangles and dotted lines indicate *Stat5* DKI cells. **e** Time course of proliferative responses of WT and *Stat5* DKI bone marrow NK cells in response to 20 ng ml^−1^ IL-15. Black squares and solid lines indicate WT cells and black triangles and dotted lines indicate *Stat5* DKI cells. **f** Time course of proliferative responses of WT and *Stat5* DKI bone marrow NK cells in response to 1000 U ml^−1^ IL-2. Black squares and solid lines indicate WT cells and black triangles and dotted lines indicate *Stat5* DKI cells. **g** Time course of proliferative responses of WT and *Stat5* DKI splenic NK cells in response to 20 ng ml^−1^ IL-15. Black squares and solid lines indicate WT cells and black triangles and dotted lines indicate *Stat5* DKI cells. **h** Time course of proliferative responses of WT and *Stat5* DKI splenic NK cells in response to 1000 U ml^−1^ IL-2. Black squares and solid lines indicate WT cells and black triangles and dotted lines indicate *Stat5* DKI cells. **i** Line graphs of Ki67 staining of WT and DKI bone marrow NK cells stimulated with 20 ng ml^−1^ of IL-15 for 0, 1, 4, 8, 24 and 48 h, as indicated. Black squares and solid lines indicate WT cells and black triangles and dotted lines indicate *Stat5* DKI cells. **j** Line graphs of Ki67 staining of WT and DKI splenic NK cells stimulated with 20 ng ml^−1^ of IL-15 for 0, 1, 4, 8, 24 and 48 h, as indicated. Black squares and solid lines indicate WT cells and black triangles and dotted lines indicate *Stat5* DKI cells. (**k** and **l**) Dose response of WT (open circles) and *Stat5* DKI (filled circles) splenic NK cells to IL-15 (**k**) or IL-2 (**l**) stimulation on day 4. The percentages of live cells were determined using live/dead cell assay kit and flow cytometry cell counting beads. Error bars in **c** and **e**−**l** are means ± SEM and statistical analyses were performed by grouped multiple *t*-test using Prism 7.0b

As noted above, there are fewer NK cells in *Stat5* DKI mice (Fig. 1h; Supplementary Fig. 2a). To evaluate their functionality, we expanded both WT and *Stat5* DKI splenic NK cells with IL-15 in vitro (Supplementary Fig. 3a) and found that both populations exhibited similar killing of ^51^Cr-labelled YAC-1 cells (Fig. 3a). Moreover, WT and *Stat5* DKI mice showed similar clearance of H2 class I gene-deficient RMA-S T lymphoma cells^45^ (Fig. 3b, c). No statistically significant difference between WT and *Stat5* DKI mice was observed in RMA-S tumour rejection experiments (Kaplan−Meier method, *p* = 0.323), although *Stat5* DKI mice appeared slightly less efficient in rejecting RMA-S tumour cells in vivo (Fig. 3d). These experiments together showed that despite there being fewer NK cells in the *Stat5* DKI mice, their cytotoxicity was normal, indicating that STAT5 dimers are sufficient for this function.

Because STAT5 tetramers are essential for normal IL-2- and IL-15-induced proliferation of CD8^+^ T cells in vitro and homoeostatic proliferation of these cells in vivo^9^, we next investigated whether the decreased NK cell numbers in *Stat5* DKI mice might result from defective proliferation. Unexpectedly, *Stat5* DKI bone marrow (Fig. 3e, f) and splenic (Fig. 3g, h) NK cells had similar or even slightly higher proliferation to IL-15 (Fig. 3e, g) or IL-2 (Fig. 3f, h). These results were consistent with a slightly higher percentage of Ki67^+^ *Stat5* DKI NK cells among freshly isolated and IL-15-stimulated bone marrow (Fig. 3i) or splenic (Fig. 3j) NK cells. Moreover, when cultured in vitro with IL-15 (Fig. 3k) or IL-2 (Fig. 3l), there were fewer viable *Stat5* DKI than WT splenic NK cells, particularly at low concentrations of the cytokines, indicating defective cytokine-induced survival of the *Stat5* DKI NK cells.

In view of the defective survival, we next examined whether there were differences in IL-15-induced gene expression by RNA-Seq. Of 467 genes that were most differentially expressed in either WT or *Stat5* DKI NK cells after 24 h of stimulation with IL-15 (RPKM ≥5, FC ≥2; Supplementary Fig. 5b, Supplementary Data 2a), 71 genes had significantly altered expression in *Stat5* DKI versus WT NK cells (Supplementary Fig. 5c, Supplementary Data 2b). Interestingly, *Ccnd1* (encoding cylin D1) was dramatically increased in response to IL-15 stimulation in *Stat5* DKI NK cells but not in WT NK cells (Supplementary Fig. 5c, d, Supplementary Data 2b). The expression of *Cdkn2b* (encoding p15^INK4B^, which forms complexes with CDK4 and CDK6 and prevents CDK activation by cyclin D) was not regulated by IL-15 in WT NK cells but its level was significantly lower in *Stat5* DKI NK cells and decreased further after IL-15 stimulation (Supplementary Fig. 5e). Conversely, *Cish*, an inhibitor of IL-15 signalling in NK cells^46^, had significantly lower expression in *Stat5* DKI NK cells stimulated by IL-15 (Supplementary Fig. 5f). Higher cyclin but lower CDK inhibitor and *Cish* expression are consistent with *Stat5* DKI NK cells exhibiting normal or even increased cell division in response to cytokine stimulation but does not explain the defective survival of these cells.

### *Bcl2* is regulated by STAT5 tetramers in NK cells

**Fig. 4:**
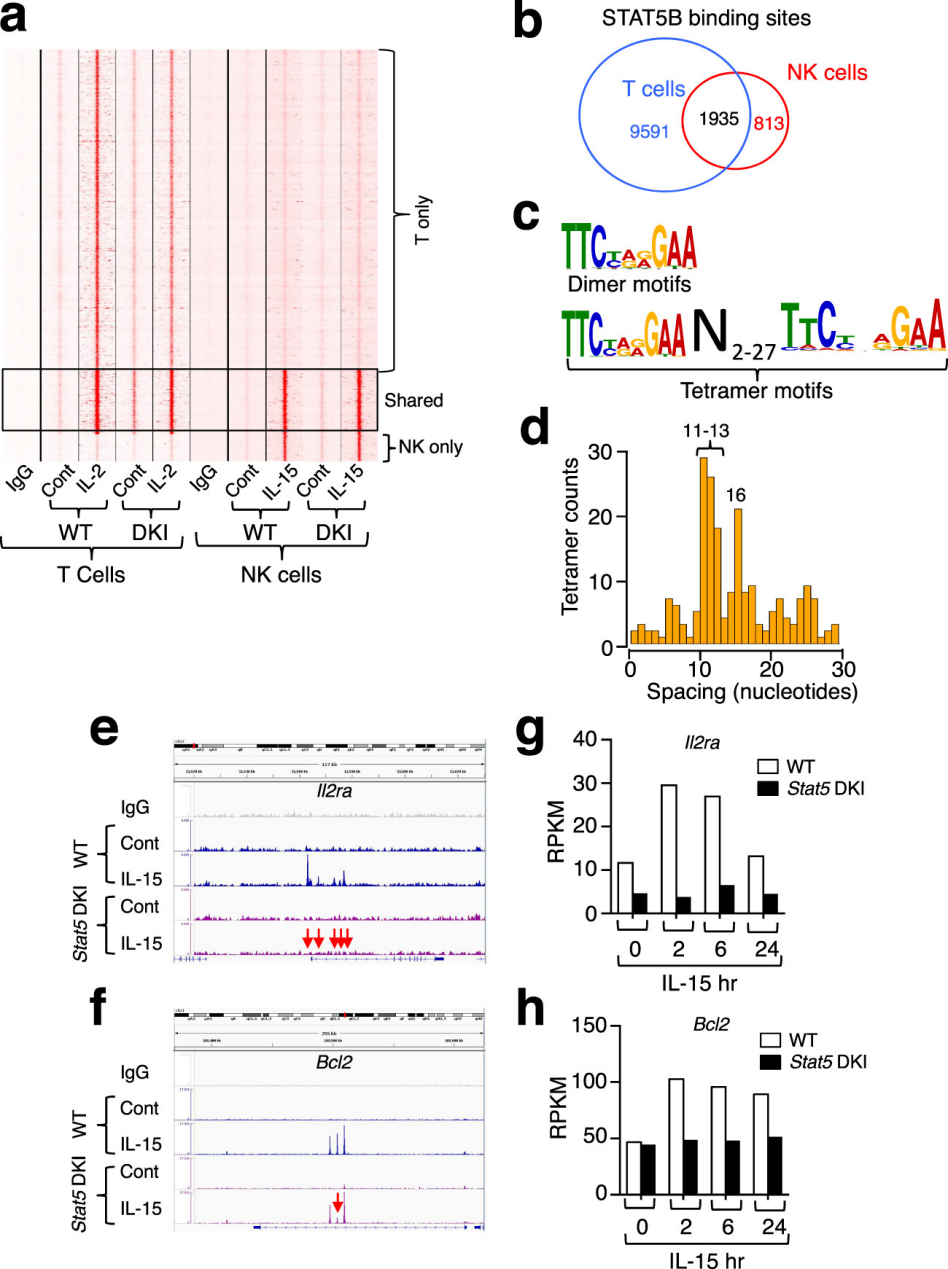
Identification of STAT5 tetramer-regulated genes. **a**, **b** Comparison of STAT5 binding sites in T and NK cells. **a** STAT5 binding sites only in T cells (T cell specific) or NK cells (NK-specific) are indicated on the right; STAT5 binding sites shared in both cells are in the black rectangle marked “Shared”. **b** Venn diagram of 9591 T-cell specific STAT5 binding sites (blue), 813 NK-specific sites (red), and 1935 sites shared in T and NK cells (black). **c** STAT5 dimer (top) and tetramer (bottom) motifs identified in NK cells. The numbers indicate potential numbers of nucleotides between two tandemly linked tetramer binding sites shown in lower panel for STAT5 tetramer motifs. **d** Spacing distribution analysis between two tandemly linked tetramer binding sites; preferred spacings between two binding sites in tetramer motifs are marked (at 11−13, and 16 nucleotides). **e** STAT5 tetramer binding in the *Il2ra* promoter and first intron. **f** STAT5 tetramer binding in the *Bcl2* intronic region (red arrows). **g** IL-15 potently induces *Il2ra* mRNAs in WT (open bars) but not in *Stat5* DKI (black bars) NK cells. **h** IL-15 potently induces *Bcl2* mRNAs in WT (open bars) but not in *Stat5* DKI (black bars) NK cells

To clarify the mechanism underlying the defective survival of *Stat5* DKI NK cells, we next sought to identify direct target genes for STAT5 dimers versus tetramers and thus performed ChIP-Seq using anti-STAT5B and WT or *Stat5* DKI splenic NK cells expanded in vitro with IL-15. We identified 2748 STAT5 binding sites in IL-15-treated WT NK cells (Fig. 4a, b), far fewer than the 11,526 sites we observed in IL-2-treated T cells (Fig. 4a, b). Approximately 70% of the sites in NK cells (1935 of 2748 sites) were shared with those in T cells, while the rest were cell type-specific (Fig. 4a, b). By comparing the STAT5 binding sites identified in WT cells (bound by STAT5 dimers and/or tetramers) with those identified in *Stat5* DKI cells (bound by STAT5 dimers but not tetramers), we identified 267 tetramer-specific binding sites; some genes had multiple sites, so these corresponded to a total 185 genes (Supplementary Data 3a). The motifs for both STAT5 dimers and tetramers (Fig. 4c) and the preferred spacing between two tandemly linked γ-interferon activated sequence (GAS) motifs in tetramer binding sites (Fig. 4d) were similar to what we defined in T cells^9^, although spacings of 11–13 and 16 bp were even more preferred in NK cells^9^ (Fig. 4d). Of the 185 genes associated with tetramer binding sites, a number of them had ≥1.5-fold lower in mRNA expression in *Stat5* DKI than WT NK cells, indicating that they were direct targets of STAT5 tetramers (Supplementary Data 3b). These included the known STAT5 tetramer-dependent *Il2ra* gene, but also *Bcl2*. STAT5 binding intensity to all of the sites in the *Il2ra* gene and to one of three intronic regions of *Bcl2* gene were significantly reduced in *Stat5* DKI cells stimulated by IL-15 (Fig. 4e, f). Consistent with the defective STAT5 tetramer binding, *Il2ra* and *Bcl2* mRNAs were induced by IL-15 in WT NK cells but not in *Stat5* DKI NK cells (Fig. 4g, h).

### Rapid cytokine depletion-induced death of *Stat5* DKI NK cells

**Fig. 5:**
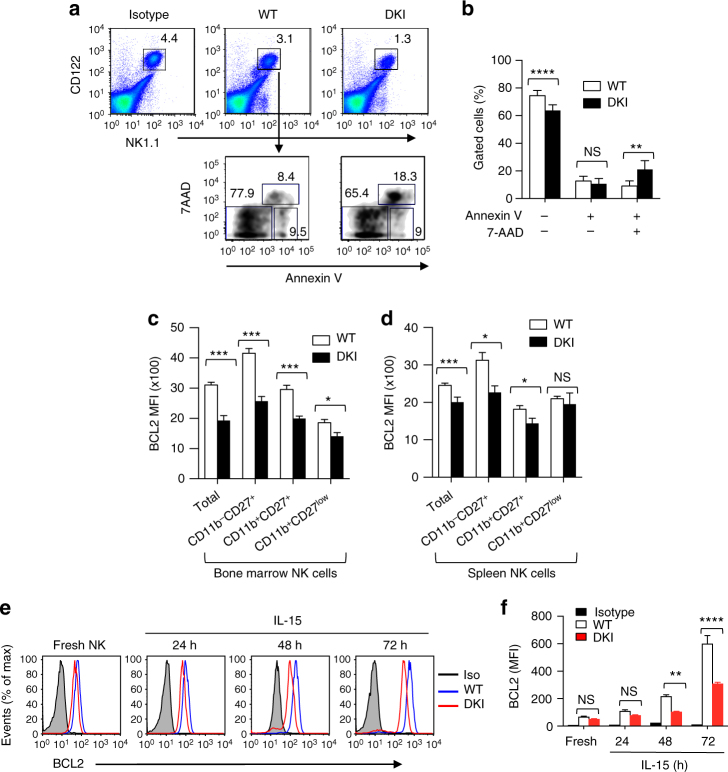
Increased apoptosis and decreased BCL2 expression as well as increased cytokine withdrawal-induced cell death in *Stat5* DKI NK cells. **a**, **b** Freshly isolated total spleen cells were stained with anti-CD3, anti-CD122, anti-NK1.1, Annexin V, and 7AAD. Apoptotic NK cells were identified as CD3^−^CD122^+^NK1.1^+^Annexin V^+^, and dead NK cells were identified as CD3^−^CD122^+^NK1.1^+^Annexin V^+^7AAD^+^ (lower panels of **a**). Percentages of apoptotic and dead cells in splenic NK cells from 7 WT (open bars) and 7 *Stat5* DKI (filled bars) mice **b**. **c**, **d** BCL2 expression (MFI) of bone marrow (**c**) and spleen (**d**) NK cells. **e** Time course of BCL2 expression in splenic NK cells cultured with 20 ng ml^−1^ IL-15; BCL2 levels were determined by flow cytometry. Isotype control antibody (shaded in grey) and BCL2 antibody staining of WT (blue) and *Stat5* DKI (red) NK cells are shown. **f** BCL2 expression levels (MFI) in IL-15-stimulated splenic NK cells from 3 WT (open bars) and 3 *Stat5* DKI (red bars) mice. The experiment was performed twice. Error bars in **b**, **c**, **d** and **f** are means ± SEM and statistical analyses were performed by grouped multiple *t*-test using Prism 7.0b

The role of STAT5 tetramers in *Bcl2* regulation and the diminished viability of *Stat5* DKI NK cells cultured with lower doses of IL-2 or IL-15 led us to further examine the viability of freshly isolated NK cells (Fig. 5a, upper panels). There was a significant increase in Annexin V^+^7AAD^+^ (Grouped Multiple *t*-test, *p* < 0.01) but not in the Annexin V^+^7AAD^−^ cells among freshly isolated splenic *Stat5* DKI NK cells as compared to WT NK cells (Fig. 5a, lower panels and Fig. 5b), consistent with the lower viability in the *Stat5* DKI NK cells. Corresponding to our RNA-Seq analysis showing significantly decreased *Bcl2* mRNA in *Stat5* DKI NK cells (Fig. 4h), BCL2 protein levels were also significantly lower in freshly isolated bone marrow (Grouped Multiple *t*-test, *p* < 0.001, Fig. 5c) and splenic (Grouped Multiple *t*-test, *p* < 0.001, Fig. 5d) *Stat5* DKI NK cells than in corresponding WT NK cells, and *Bcl2* mRNA (Fig. 4h, Supplementary Data 2a) and BCL2 protein (Fig. 5e, f) were less potently induced by IL-15 in *Stat5* DKI NK cells than in WT NK cells.

**Fig. 6:**
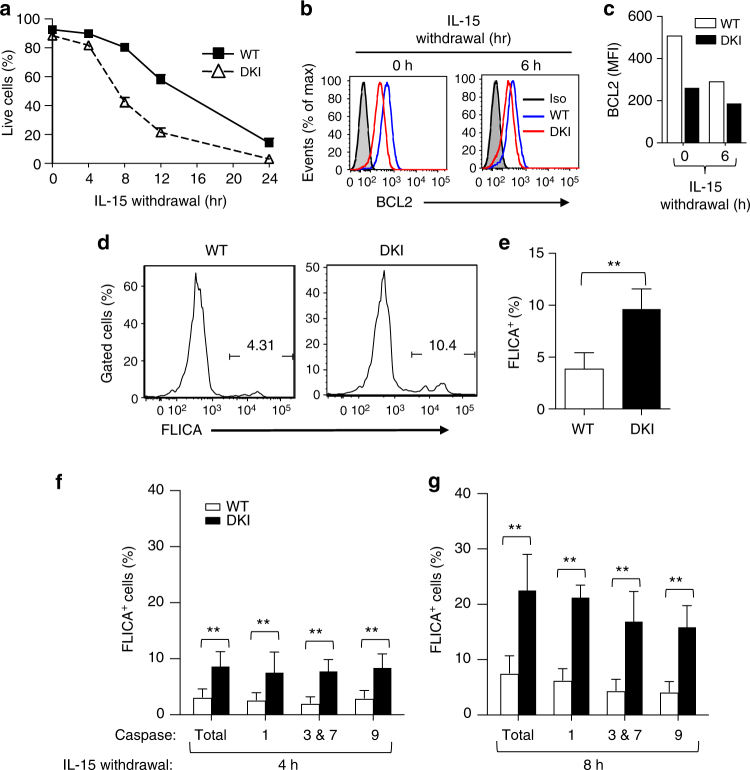
Dysregulated expression of cell survival-related genes and increased active caspase levels in *Stat5* DKI NK cells. **a** Time course of the percentage of viable WT (filled squares) or *Stat5* DKI (open triangles) NK cells following IL-15 withdrawal. Cells were isolated from spleen and cultured in vitro with IL-15 for 6 days prior to withdrawal. Three WT and *Stat5* DKI mice were used. The experiment was performed twice. Error bars are means ± SEM and statistical analyses were performed by grouped multiple *t*-test using Prism 7.0b. **b** Histograms of intracellular BCL2 staining of WT (blue) and *Stat5* DKI (red) NK cells. Staining was of cells cultured in 20 ng ml^−1^ IL-15 (0 h) or 6 h upon IL-15 withdrawal. **c** Summary of BCL2 levels (MFI) before (0 h) and after IL-15 withdrawal for 6 h. **d** Representative FLICA staining of freshly isolated splenic NK cells. The numbers in the gated regions are FLICA^+^ NK cells (%). **e** Summary of **d** of freshly isolated splenic WT (open bar) and *Stat5* DKI NK (filled bar) cells analysed using the FLICA Poly Caspase Assay kit. Error bars are means ± SEM and statistical analyses were performed by grouped multiple *t*-test using Prism 7.0b. **f** Summary of FLICA staining of WT (open bars) and *Stat5* DKI (closed bars) NK cells at 4 h after IL-15 withdrawal for poly caspases (Total), caspase-1 (1), caspases-3 and 7 (3, 7), and caspase-9. Error bars are means ± SEM and statistical analyses were performed by grouped multiple *t*-test using Prism 7.0b. **g** Summary of FLICA staining of WT (open bars) and *Stat5* DKI (closed bars) NK cells at 8 h after IL-15 withdrawal for poly caspases (Total), caspase-1 (1), caspases-3, 7 (3, 7), and caspase-9. Error bars are means ± SEM and statistical analyses were performed by grouped multiple *t*-test using Prism 7.0b

The lower induction of *Bcl2* expression in the *Stat5* DKI NK cells prompted us to evaluate the effect of IL-15 withdrawal. Importantly, *Stat5* DKI NK cells that were expanded in vitro with IL-15 exhibited significantly more rapid cell death than we observed with similarly treated WT NK cells (Fig. 6a). BCL2 protein levels (Fig. 6b, c) also were lower in *Stat5* DKI than in WT NK cells after IL-15 withdrawal, consistent with the increased apoptosis of *Stat5* DKI NK cells and elucidating the basis for the lower number of NK cells in *Stat5* DKI mice. Because BCL2 supports cellular viability and decreasing its expression can initiate an apoptotic cascade by activating caspases and eventually leading to cell death^47,48^, we hypothesised that decreased expression of anti-apoptotic factors rather than the induction of pro-apoptotic protein(s) was the basis for increased NK cell death in the *Stat5* DKI mice. We next assessed active caspase levels using the fluorochrome-labelled inhibitors of caspases (FLICA) assay and found significantly increased FLICA^+^ cells in freshly isolated splenic *Stat5* DKI NK cells (Grouped Multiple *t*-test, *p* < 0.01, Fig. 6d, e) and following IL-15 withdrawal from *Stat5* DKI NK cells that were cultured in vitro with IL-15 (Grouped Multiple *t*-test, *p* < 0.01, Fig. 6f, g). These data further establish the model that lower BCL2 expression in *Stat5* DKI NK cells promotes caspase activation and the apoptosis/death of these cells.

## Discussion

**Fig. 7:**
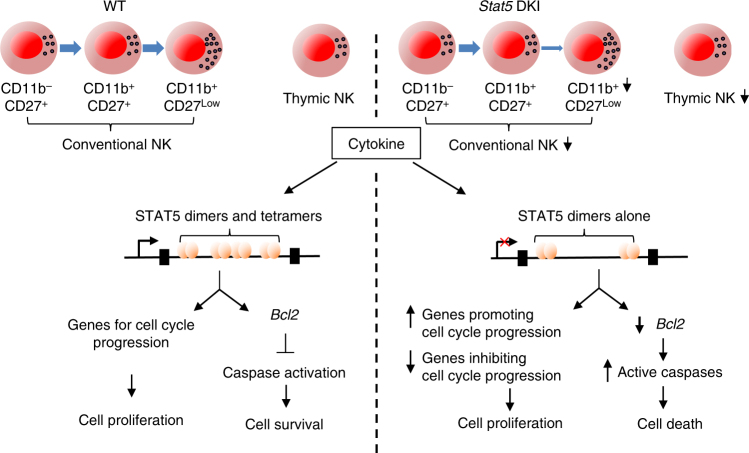
Schematic of NK cell maturation, proliferation and survival in WT versus *Stat5* DKI mice. WT mice (on the left) exhibit normal maturation, proliferation and survival of conventional NK cells and thymic NK cell cells. *Stat5* DKI mice, which lack STAT5 tetramers (on the right), have defective NK cell maturation with decreased numbers of conventional NK cells as well as decreased numbers of thymic NK cells; their NK-cell proliferation is not defective, but *Bcl2* expression is decreased and active caspases are increased, resulting in increased NK cell death

Formation of STAT1, STAT4 and STAT5 tetramers was demonstrated years ago^5–8^, but the in vivo roles of STAT tetramers were only recently elucidated, based on the analysis of *Stat5a* and *Stat5b* DKI^9^ and *Stat1* knockin (KI)^49^ mice, and recently a model of the three-dimensional structure of STAT5 tetramers was generated that helps to explain their observed binding preferences^50^. STAT5 tetramers are required for maintaining normal numbers of peripheral CD8^+^ T cells, CD4^+^CD25^+^ T cells, and NK cells, proliferative response of T cells to IL-2 or IL-15 stimulation, and T regulatory (Treg) cell function in vivo, but they are not required for viability, organ development, B cell development and function, or for the normal numbers of CD4^+^ T cells^9^. In this study, we have demonstrated that STAT5 dimers are sufficient for early NK development, whereas STAT5 tetramers were required for later natural killer (NK) cell maturation (Fig. 7). Notably, in *Stat5* DKI mice, the frequency of CD11b^−^CD27^+^ cells was increased, whereas the more mature CD11b^+^CD27^low^ NK cells were nearly absent in bone marrow and significantly decreased in spleen. Analysis of RNA-Seq data from WT NK cells revealed that most of the genes known to be critical for NK cell development were expressed similarly during the CD11b^−^CD27^+^ to CD11b^+^CD27^+^ or CD11b^+^CD27^+^ to CD11b^+^CD27^low^ NK cell transitions, indicating their relatively constant expression during NK cell development. Interestingly, however, we identified 892 differentially expressed genes during the CD11b^−^CD27^+^ to CD11b^+^CD27^+^ and/or the CD11b^+^CD27^+^ to CD11b^+^CD27^low^ NK cell transition, implicating them as playing roles in the NK cell maturation process. Among these differentially expressed genes, a subset had dysregulated expression in the corresponding subpopulations of *Stat5* DKI NK cells. Interestingly, three genes (*Mki67*, *Ccna2* and *Ccnb2*) that are involved in proliferation/cycling were downregulated in WT but not in *Stat5* CD11b^+^CD27^low^ NK cells.

In contrast to the defects in cell cycle progression of STAT5 tetramer-deficient T cells in response to high dose IL-2 or IL-15 stimulation in vitro or homoeostatic proliferation in vivo, STAT5 dimers were sufficient for cell cycle progression of NK cells in response to IL-2 or IL-15. In fact, *Stat5* DKI NK cells exhibited slightly faster cell division in response to high doses of these cytokines, and these cells expressed more Ki67^+^ cells than did WT NK cells. Interestingly, as compared to WT NK cells, *Stat5* DKI NK cells had similar or increased expression levels of *Ccn* family genes, similar expression of most *Cdk*, *Cdkn* and *Chek* family genes (Supplementary Data 2b, c), but decreased expression of *Cdkn2b*, *Cdkn1a* and *Cish* (Supplementary Data 2b). Thus, unlike their essential roles in IL-2-induced T-cell proliferation, STAT5 tetramers are not required for NK cell cycle progression.

Although *Stat5* DKI CD8^+^ T cells^9^ and NK cells both exhibit increased cell death upon cytokine withdrawal, NK cells showed more rapid and enhanced death upon cytokine withdrawal, and this correlated with decreased expression of anti-apoptotic proteins in these cells (Fig. 7). Importantly, active caspases were also increased in *Stat5* DKI NK cells, consistent with decreased expression of anti-apoptotic protein BCL2 triggering the activation of caspases, especially the initiator caspases, caspase-1 and caspase-9, which can then activate downstream effector molecules involved in cell death. Interestingly, in addition to the IL-15 → STAT5 → BCL2 survival pathway, it was reported that the IL-15 → ERK → FOXO3A pathway augments expression of MCL1, an anti-apoptotic BCL2 family protein, but diminishes expression of pro-apoptotic BIM (*Bcl2l11*) and NOXA (*Pmaip1*), which would promote the survival of WT NK cells^51^. Given that there is lower expression of *Bcl2l11* mRNA in *Stat5* DKI NK cells (Supplementary Data 2a), similar expression of *Mcl1* mRNA in *Stat5* DKI and WT NK cells (Supplementary Data 2c), and very low expression of *Pmaip1* (Reads Per Kilobase of transcript per Million mapped reads <3) in both WT and *Stat5* DKI NK cells in response to IL-15 stimulation, MCL1, BIM, and NOXA do not appear to contribute to increased death in the *Stat5* DKI NK cells. Instead, our data support the concept that the absence of STAT5 tetramers lowers survival primarily due to diminished BCL2 and augmented caspase activity.

Interestingly, STAT5 tetramer formation was reported to be associated with leukaemogenesis in a mouse model^52^. However, a caveat is that the study was performed in part with the STAT5A W37A mutant, which initially was reported to be important for tetramerization^7^ but then was shown to affect stability of the protein rather than tetramer formation^6^. Thus, further work is needed to better define the potential relationship of STAT5 tetramers to leukaemogenesis. Our *Stat5* DKI mice may allow one to better evaluate the funcitons of STAT5 tetramers in the development of leukaemia.

In summary, our data underscore non-redundant roles of STAT5 dimers and tetramers in the development, maturation, survival, and expansion of mouse NK cells, with STAT5 dimers being critical for early development of conventional NK cells and their expansion in vitro, whereas STAT5 tetramers are essential for later NK maturation, expansion at lower concentration of the cytokines, and survival, providing an explanation for the lower NK cell numbers in *Stat5* DKI mice. We demonstrate that STAT5 tetramers are required for normal *Bcl2* expression, with little if any IL-15-induced expression of *Bcl2* in *Stat5* DKI NK cells. Moreover, the lack of STAT5 tetramers is associated with the activation of caspases to initiate the cascade for apoptosis/cell death, underscoring the role of STAT5 tetramers in the survival as well as the maturation of NK cells. Finally, our study also implies that mutation(s) of the STAT5 N-domain that diminish or abolish tetramer formation may result in NK cells deficiency, and interfering with tetramer formation by small molecule inhibitors could potentially be a mechanism for controlling NK cell numbers in NK leukaemia.

## Methods

### Mice

*Stat5* DKI mice were described previously^9^. Both female and male mice from 8 to 20-weeks old were used for the experiments. The mice were housed in specific pathogen-free mouse facilities at National Institutes of Health (NIH) Bethesda campus. All mouse protocols were approved by the National Heart, Lung and Blood Institute Animal Care Use Committee, and experiments followed NIH guidelines for using animals in intramural research.

### Flow cytometric analyses

For cell surface marker staining, 10^6^ cells in 100 μl PBS containing 0.5% BSA and 0.1% sodium azide were stained with 0.5 μg of fluorescent-labelled monoclonal antibodies (BD Biosciences, San Jose, CA or BioLegend Inc., San Diego, CA) for CD3 (145-2C11), CD122 (TM-β1, IL-2Rβ), NK1.1 (PK136), CD11b (M1/70), CD27 (LG.3A10), CD49b (DX5), Ly49A (A1), Ly49H (3D10); for detection of apoptosis, the cells were additionally stained with Annexin V (5 μl per sample), 7AAD (5 μl per sample); for BCL2 expression, after fixation and permibilization the cells were stained with 20 μl of FITC- or PE-labelled hamster anti-mouse BCL2 Set (3F11, BD Biosciences); for exclusion of linage-positive populations, 10^6^ cells were first stained with 0.5 μg of biotin-labelled antibodies (BioLegend) for TCRβ (H57-597), CD3 (145-2C11), CD4 (H129.19), CD8a (53−6.7), CD19 (1D3), IgM (RMM-1), Ter119 (Ter119), and then with 0.125 μg fluorescent-labelled streptavidin (BioLegend).

To measure caspase activity in the cells, total splenocytes were incubated at 37 °C for 1 h with fluorescent-labelled caspase inhibitor FAM-VAD-FMK probes (FLICA reagent) for Poly caspases (91), caspase-1 (97), caspases-3, 7 (93), and caspase-9 (912) according to the manufacturer’s instructions (Immunochemistry Technologies, Bloomington, MN, USA). Data were acquired using a FACSCanto II flow cytometer (BD Immunocytometry Systems) and analysed using FlowJo (v9.7.5, Tree Star, Inc., Ashland, OR).

### Cell sorting and RNA isolation

To sort splenic NK subpopulations, total splenocytes were isolated from 10 WT or 15 *Stat5* DKI female mice and total NK cells were enriched via negative selection using antibodies (0.5 μg per 10^6^ cells) for IgM, CD19, TCRβ, CD3, CD4, CD8, Ter119 (Biolegend) and Dynabeads Sheep anti-Rat IgG (2.5 μl per 10^6^ cells, Invitrogen, Grand Island, NY). CD3^-^CD122^+^NK1.1^+^ NK cells were then sorted on a FACSAriaII cell sorter for CD11b^−^CD27^+^, CD11b^+^CD27^+^ and CD11b^+^CD27^low^ NK subpopulations. Sorted cells were washed with PBS once, lyzed in Trizol Reagent (Invitrogen, Grand Island, NY), and total RNA was isolated using Direct-zol RNA MiniPrep Kit (R2050, Zymo Research, Irvine, CA).

### Cell proliferation and survival assays

To monitor cell division, 20 million splenic or bone marrow cells from WT or *Stat5* DKI mice were labelled in 1 ml of PBS with 2.5 μM of CFSE (CellTracer CFSE Cell Proliferation Kit, Invitrogen, Carlsbad, CA) at room temperature for 7 min, washed once with serum and twice with complete RPMI-1640 medium, 1.5 × 10^6^ ml^−1^ CFSE-labelled cells were then cultured in the presence of 20 ng ml^−1^ recombinant human IL-15 (R&D Systems, Minneapolis MN or BioLegend), and fresh rhIL-15 was added every 2 days. NK cell division was monitored for CFSE dilution using flow cytometry on day 2, 3 and 4 by staining cells with fluorescent-labelled antibodies for CD3, CD122 and NK1.1.

To determine cell viability after IL-15 withdrawal, column purified (Miltenyi Biotec Inc., San Diego, CA) NK cells from WT or *Stat5* DKI mice were cultured in complete RPMI-1640 medium supplemented with 20 ng ml^−1^ rhIL-15 for 6–7 days, fresh rhIL-15 was added every 2 days, cells were then washed three times with complete medium, cultured in complete medium without IL-15, stained at the indicated time points, and viable NK cells were determined as CD3^−^CD122^+^NK1.1^+^Annexin V^−^7AAD^−^. Annexin V^+^7AAD^−^ cells were scored as apoptotic cells and Annexin V^+^7AAD^+^ as dead cells.

### Cell cytotoxicity assays

For in vitro NK cell cytotoxicity assays, splenic NK cells from 3 of WT and *Stat5* DKI mice were enriched using NK cell negative selection kit (Miltenyi Biotech, San Diego, CA) and cultured with 20 ng ml^−1^ rhIL-15 (R&D) for 6 days to reach similarly high purity of NK cells, incubated at indicated ratio in triplicates with ^51^Cr-labelled YAC-1 cells at 37 °C for 4 h. Specific target (YAC-1) cell lysis was calculated.

For in vivo NK cell cytotoxicity assays, H2 class I gene-deficient RMA-S and H2 class I gene expressing RMA cells (provided by Dr. Lewis L. Lanier, University of Califonia, San Francisco) were labelled by 0.5 and 5 μM of CFSE, respectively, equal number of the labelled RMA-S and RMA cells were mixed, and 2 × 10^6^ cells were intraperitoneally injected into each of WT and *Stat5* DKI mice. Peritoneal cells were recovered 16 h after injection and the recovery rate of each cell types was determined by flow cytometry.

### Tumour rejection experiments

RMA-S cells were cultured in complete RPMI-1640 medium supplemented with 50 μM β-mercaptoenthol. For tumour rejection experiments, the cells were washed with PBS 3 times, resuspended in PBS, and 2 × 10^5^ cells in 150 μl PBS were subcutaneously injected into the scruff of the neck of each mouse. Tumour size was measured using a digital caliper daily 7 days after injection. Animals were killed when the tumour reached 20 mm in diameter or showed severe ulceration.

### RNA-Seq library preparation and sequencing

Libraries were prepared using 150 ng of total RNA from bone marrow NK cells and splenic NK subpopulations and KAPA Stranded RNA-Seq Library Preparation Kit (KK8400, KAPA Biosystems, Wilmington, MA), and each library was indexed using NEXflex DNA Barcodes-24 (NOVA-514103, BIOO Scientific, Austin, TX). Barcoded PCR products were purified on 2% E-Gel, 250–400 bp fragments were purified, quantified on Qbit (Invitrogen), mixed, and sequenced on an Illumina HiSeq 2500 or HiSeq 3000 platform (Illumina, San Diego, CA).

### ChIP-Seq library preparation and sequencing

Splenic NK cells from 10 WT and 13 *Stat5* DKI mice were purified (Miltenyi Biotec Inc.), expanded in vitro with 20 ng ml^−1^ of human recombinant IL-15 (R&D Systems or BioLegend) for 10 days, with fresh rhIL-15 being added every two days, yielding 50–80 million of nearly 100% pure CD3^−^CD122^+^NK1.1^+^ cells. The cells were washed three times with medium, rested in complete RPMI-1640 medium without IL-15 for 2 h, not treated or treated with 40 ng ml^−1^ of IL-15 for 1 h, and cross-linked with 1% formaldehyde (methanol-free, Pierce, Rockford, IL) at room temperature for 10 min. After sonication, fragmented chromatin equivalent to 10 million cells were immunoprecipitated with control rabbit IgG (sc-3888, Santa Cruz Biotechnology, Dallas TX) or anti-STAT5B (AF1584, R&D Systems) and Magna ChIP Protein A + G Magnetic Beads (16–663, Millipore, Billerica MA). ChIP-Seq DNA libraries were prepared using KAPA LTP Library Preparation Kit (KK8232, Kapa Biosystems, Wilmington, MA) and NEXflex DNA Barcodes-24 (NOVA-514103, BIOO Scientific, Austin, TX), and the libraries were then sequenced on an Illumina HiSeq 3000 platform.

### RNA-seq analysis

Sequenced reads (50 bp, single end) were obtained with the Illumina CASAVA pipeline and mapped to the mouse genome (mm9/NCBI37) using TopHat 2.0.11. Only uniquely mapped reads were retained. RefSeq gene database was downloaded from the UCSC genome browser for RNA-Seq analysis. Raw counts that fell on exons of each gene were calculated and normalised to obtain RPKM (Reads Per Kilobase per Million mapped reads) values. Differentially expressed genes were identified based on indicated RPKM and fold change thresholds. The expression heatmaps were generated with the “pheatmap” library in R using the scale = “row” parameter. Scatter plots are generated by DataGraph 4.1 (Visual Data Tools, Inc.)

### ChIP-seq analysis

Sequenced reads were aligned to the mouse genome (mm9/NCBI37) using Bowtie 0.12.9. Only uniquely mapped reads were retained. Normalised read counts were summed in 20 bp sliding windows and displayed in the Integrative Genomics Viewer (IGV). MACS 1.4.2 was used to call binding sites (peaks) relative to control libraries. The *p*-value threshold was set as 1 × 10^−5^, and the effective genome size was set as 2.7 × 10^9^. Transcription factor was considered as ‘bound’ to genes if peaks were within 5 kb upstream of the transcription start site and anywhere across the gene body.

To compute STAT5B binding profiles in T and NK cells, we chose ±3 kb regions around combined peak summits and divided the regions into bins of 20-bp windows. Reads (or tags) that fell into each bin were counted and normalised by library size. Heatmaps of binding profiles were generated based on K-means clustering and plotted using seqMiner 1.3.3. STAT5 dimer and tetramer motifs were identified as described previously^9^.

### Statistical analysis

Statistical analyses were performed by grouped multiple *t*-test analysis using Prism 7.0b (GraphPad Software, Inc., La Jolla, CA), error bars are means ± SEM; ****p < 0.0001; ***p < 0.001, **p < 0.01; *p < 0.05; NS, *p* > 0.05.

### Data availability

ChIP-Seq and RNA-Seq data have been deposited in the National Center for Biotechnology Information Gene Expression Omnibus (GEO) database (https://www.ncbi.nlm.nih.gov/geo) under the GEO Series accession numbers GSE101470 and GSE36890. All other materials are available from the corresponding authors on request.

## Electronic supplementary material

Supplementary Information

Description of Additional Supplementary Files

Supplementary Data 1

Supplementary Data 2

Supplementary Data 3
