# Publisher Correction: Critical functions for STAT5 tetramers in the maturation and survival of natural killer cells

**DOI:** 10.1038/s41467-025-56265-3

**Published:** 2025-01-21

**Authors:** Jian-Xin Lin, Ning Du, Peng Li, Majid Kazemian, Tesfay Gebregiorgis, Rosanne Spolski, Warren J. Leonard

**Affiliations:** 1 Laboratory of Molecular Immunology and the Immunology Center, National Heart, Lung, and Blood Institute, National Institutes of Health, Bethesda, MD 20892-1674 USA; 2 Present Address: Department of Biochemistry and Computer Science, Purdue University, West Lafayette, IN 47906 USA

Correction to: *Nature Communications* 10.1038/s41467-017-01477-5, published online 06 November 2017

The original version of this Article contained an error in Fig. 4a, in which the heatmap graph for track 3 (T Cells-WT-IL-2) and track 4 (T Cells-DKI-Cont) were swapped inadvertently; similarly, track 8 (NK-WT-IL-15) and track 9 (NK-DKI-Cont) were also swapped erroneously. This has been corrected in both the PDF and HTML versions of the Article.

