# Supplementary material for: Critical functions for STAT5 tetramers in the maturation and survival of natural killer cells
Source: Nat Commun. 2017 Nov 6;8:1320. doi: 10.1038/s41467-017-01477-5 (PMC5673064; doi:10.1038/s41467-017-01477-5)
Supplement: Supplementary file 1 — Supplementary Information [file 41467_2017_1477_MOESM1_ESM.pdf]

Supplementary Figure 1

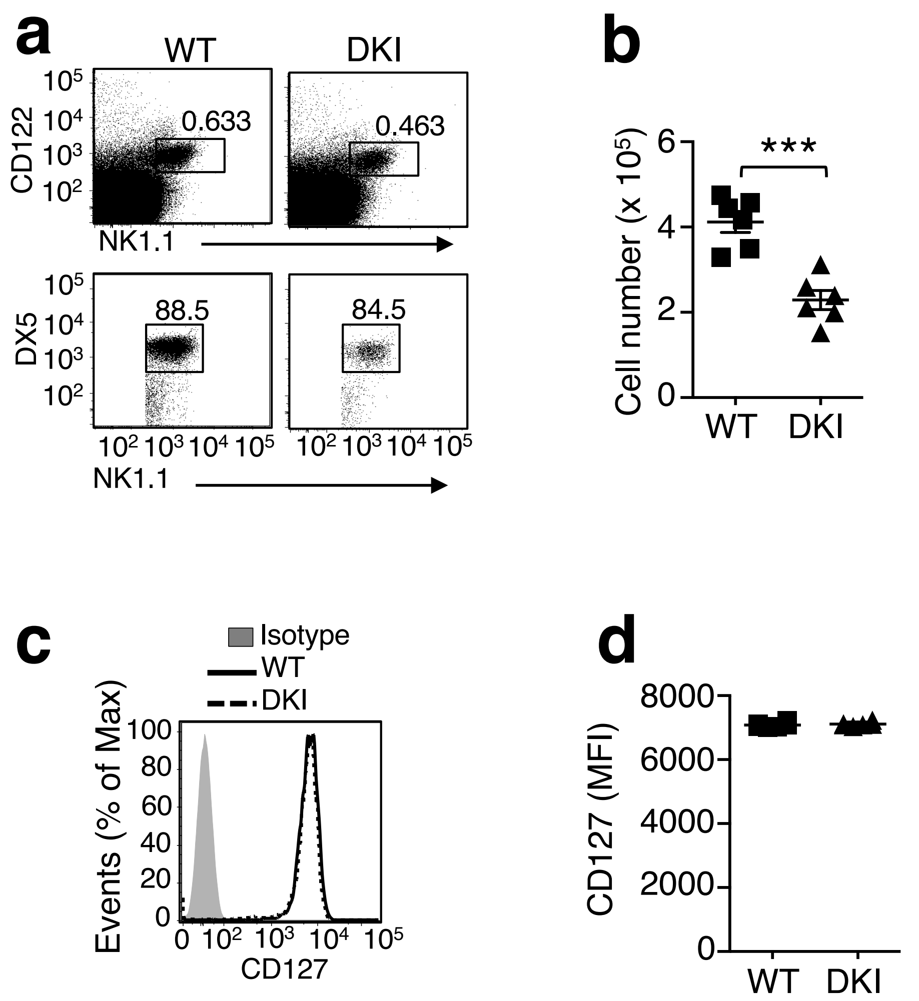

Supplementary Figure 1. Decreased thymic NK cells in *Stat5* DKI mice. (**a**) Flow cytometric analysis of total thymic NK cells (lin [TCRβCD3CD4CD8CD19Ter119]$^{-}$ CD122^{+}) (upper panels) and lin^{-}CD122^{+}NK1.1^{+}DX5^{+} mature NK cells (lower panels) in WT and *Stat5* DKI thymus. (**b**) Total thymic NK cell numbers from 6 WT (black squares) and 6 *Stat5* DKI (black triangles) mice. The experiment was performed twice, each with 3 WT and 3 DKI mice; shown are representative mice. The differences in the thymic NK cell numbers between WT and *Stat5* DKI mice were significant (Multiple *t* test, $p < 0.001$). (**c**) Representative flow cytometric profiles for CD127 expression on *Stat5* DKI (dotted line) and WT (solid line) thymic NK cells. (**d**) Summary of CD127 levels (MFI) between WT (black squares) and DKI (black triangles) thymic NK cells. Error bars are means ± SEM.

Supplementary Figure 2

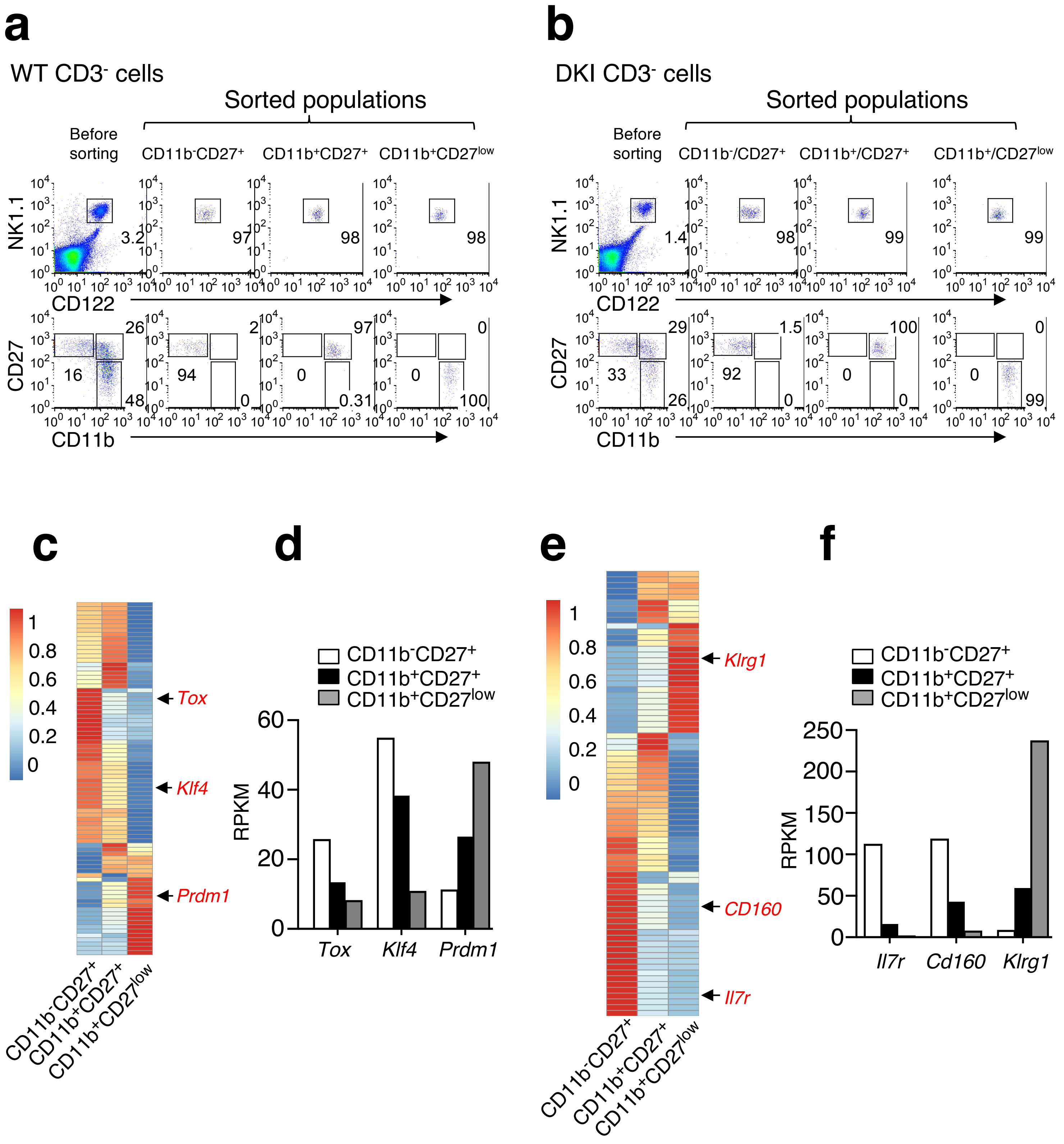

Supplementary Figure 2. Differentially expressed genes encoding transcription factors and receptors in WT CD11b^{-}CD27^{+}, CD11b^{+}CD27^{+}, and CD11b^{+}CD27^{low} subpopulations of NK cells. (**a**) Flow cytometric profiles of splenic NK cells before and after sorting from WT mice. (**b**) Flow cytometric profiles of splenic NK cells before and after sorting from *Stat5* DKI mice. (**c**) Heat maps of mRNAs for transcription factors differentially expressed in WT NK subpopulations. (**d**) Expression (RPKM) shown for three transcription factors (*Tox*, *Klf4*, and *Prdm1*) known to be important for NK cell development and/or maturation. (**e**) Heat maps of mRNAs for cytokines, chemokines, their receptors, NK receptors, and other receptors differentially expressed in WT NK subpopulations, (**f**) Expression (RPKM) shown for a cytokine receptor (*Il7r*) and two NK receptors (*Cd160* and *Klrg1*).

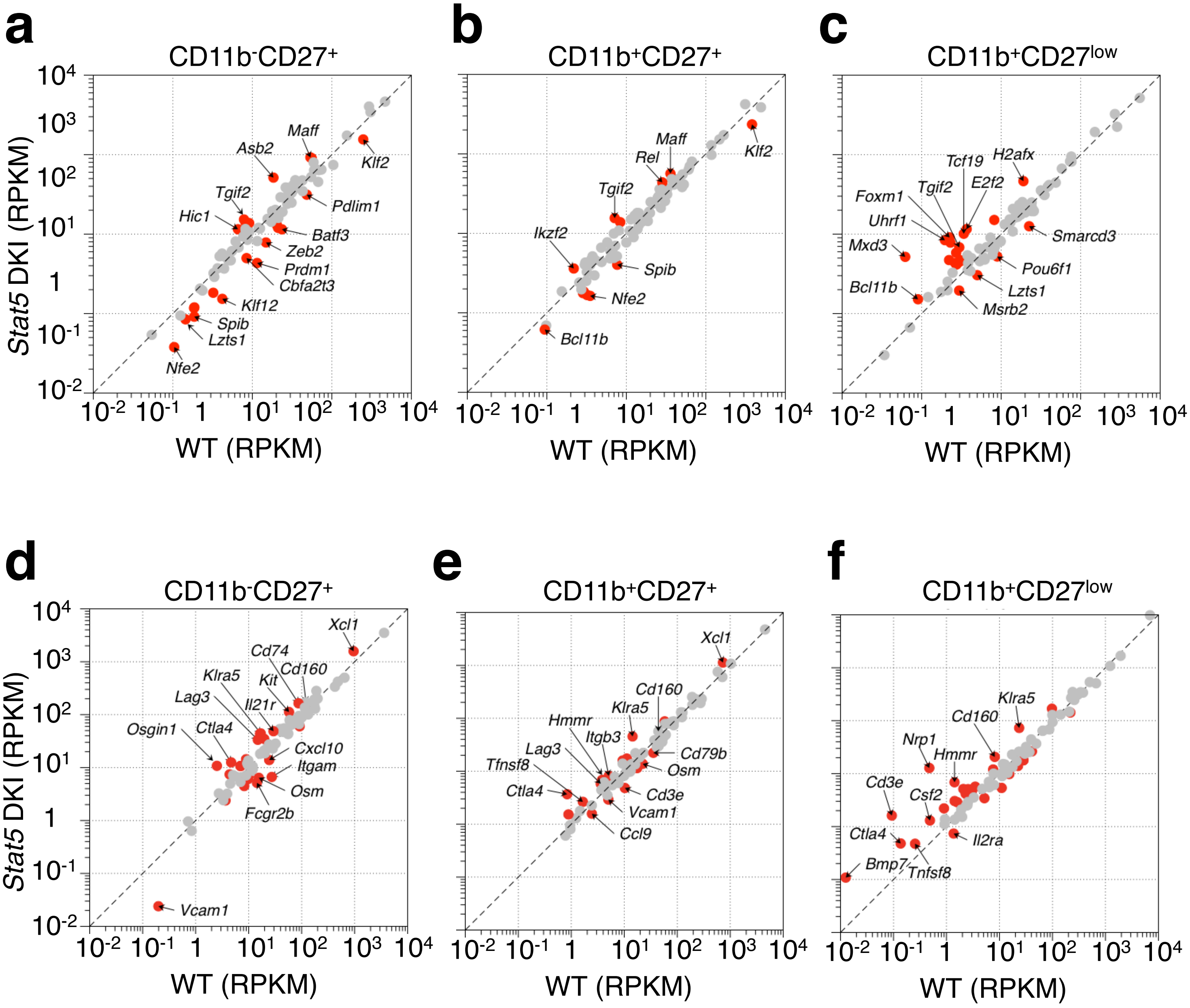

Supplementary Figure 3. Altered expression of genes encoding transcription factors and receptors in CD11b$^-$CD27$^+$, CD11b$^+$CD27$^+$, and CD11b$^+$CD27^{low} subpopulations of *Stat5* DKI NK cells. (**a**-**c**) Scatter plots of genes encoding transcription factors whose expression were altered (in solid red circles) in CD11b$^-$CD27$^+$ (**a**), CD11b$^+$CD27$^+$ (**b**), and CD11b$^+$CD27^{low} (**c**) subpopulations of *Stat5* DKI NK cells. (**d**-**f**) Scatter plots of genes encoding receptors whose expression were altered (in solid red circles) in CD11b$^-$CD27$^+$ (**d**), CD11b$^+$CD27$^+$ (**e**), and CD11b$^+$CD27^{low} (**f**) subpopulations of *Stat5* DKI NK cells.

Supplementary Figure 4

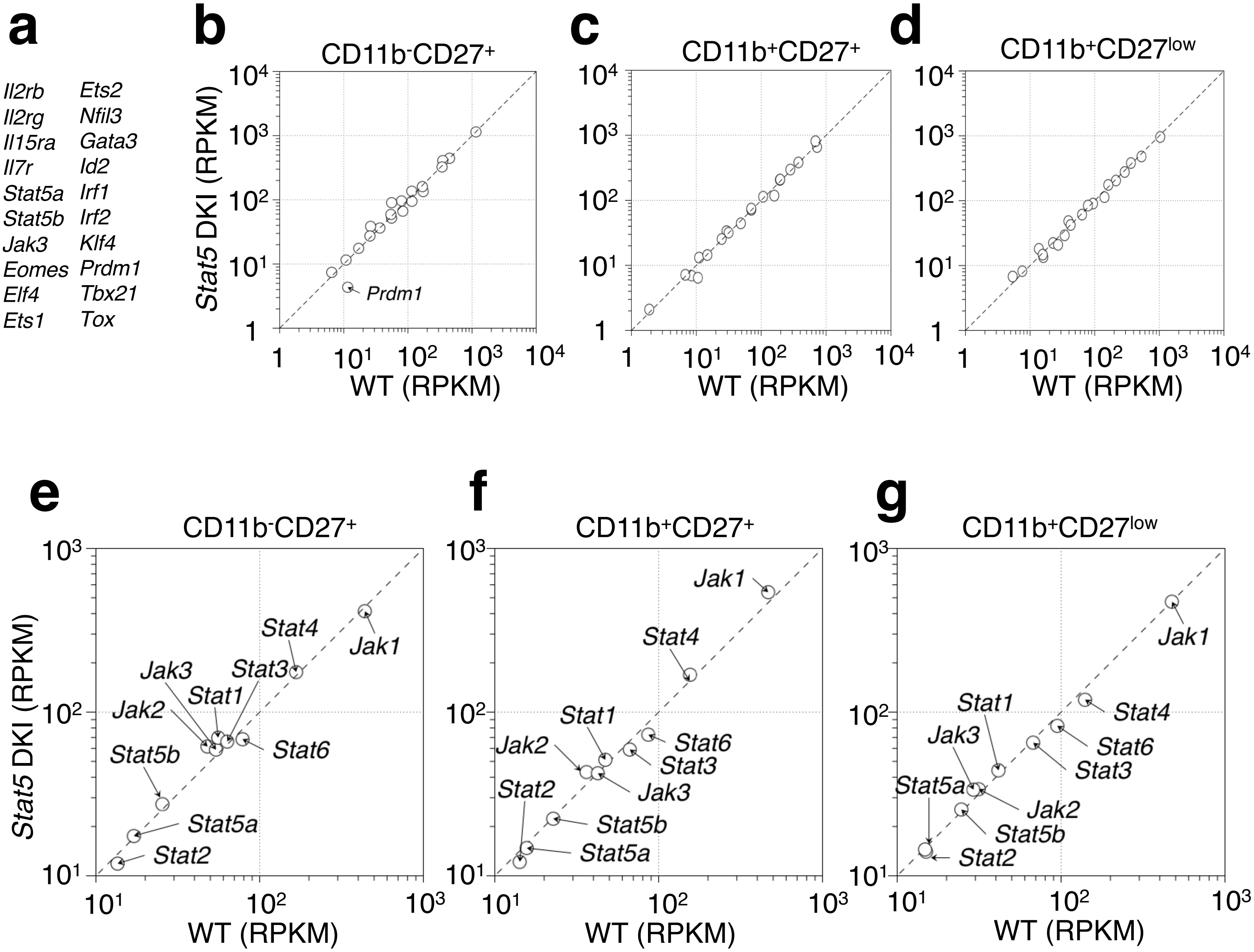

Supplementary Figure 4. **Expression profile of genes that are known to be critical for NK cell development and those encoding JAK kinases and STAT proteins**. (**a**) List of genes that are known to be critical for NK cell development and maturation. (**b-d**) Scatter Plots showing generally similar expression of the genes in panel **a** in WT and *Stat5* DKI CD11b$^-$CD27$^+$ (**h**), CD11b$^+$CD27$^+$ (**i**), and CD11b$^+$CD27^{low} (**j**) NK cells. **(e-g**) Scatter plots of genes encoding JAK kinases and STAT proteins in CD11b$^-$CD27$^+$ (**e**), CD11b$^+$CD27$^+$ (**f**), and CD11b$^+$CD27^{low} (**g**) subpopulations of *Stat5* DKI and WT NK cells.

Supplementary Figure 5

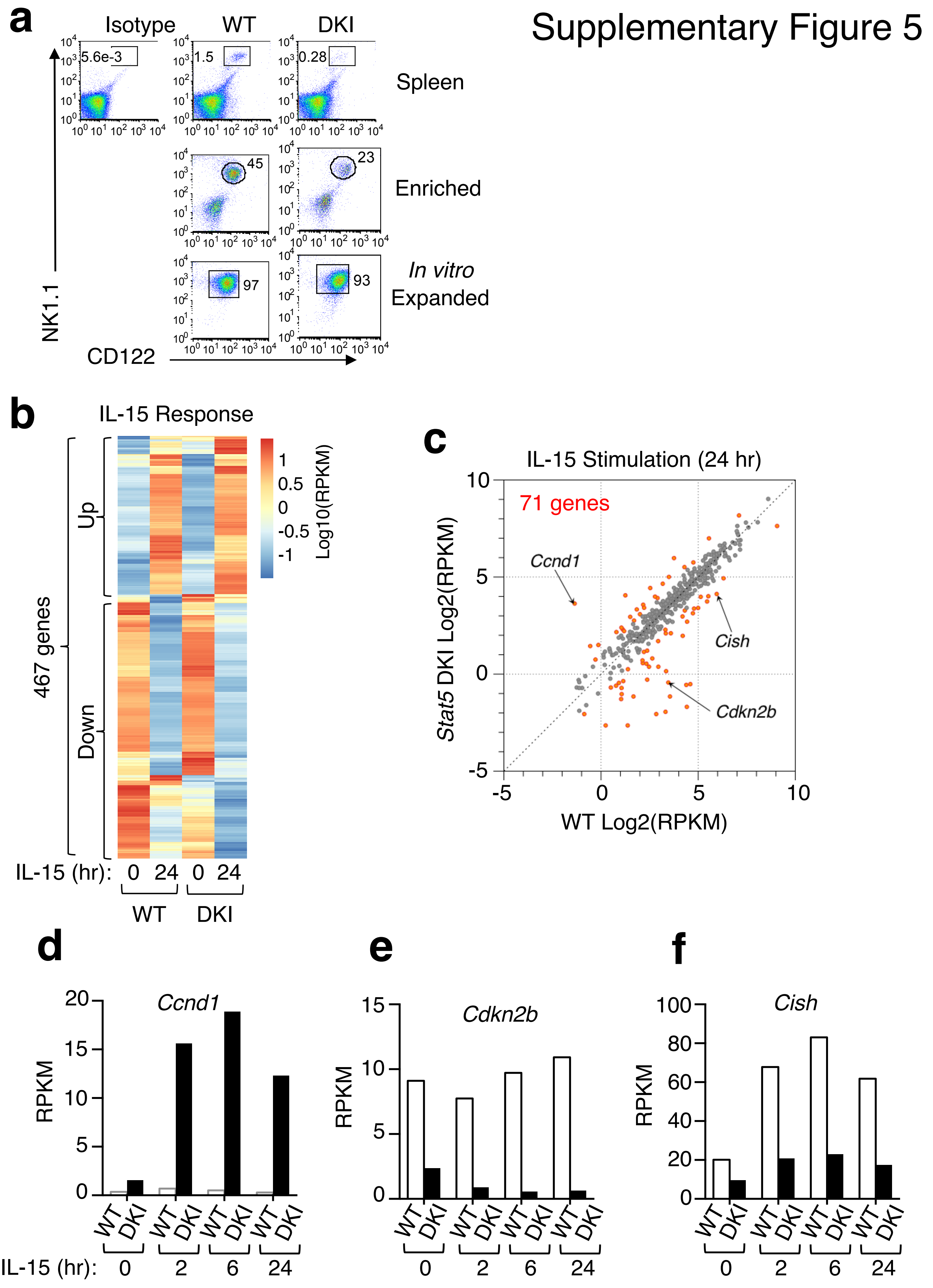

Supplementary Figure 5. Gene expression profiles of WT and *Stat5* DKI NK cells in response to IL-15 stimulation. **(a)** Splenic NK cells were isolated and purified from WT and *Stat5* DKI mice. NK cells ($CD3^-CD122^+NK1.1^+$; top and middle rows) were expanded *in vitro* by culturing with IL-15 for 6 days (bottom row). (**b**) Heat map showing genes regulated by IL-15 in WT and *Stat5* DKI NK cells, with 467 genes up- or down-regulated in WT NK cells in response to IL-15, as indicated by black brackets on the left. The expression of some genes was altered in *Stat5* DKI NK cells. (**c**) Scatter plot showing 71 genes (red circles) whose expression was altered in *Stat5* DKI NK cells stimulated by IL-15 for 24 hr. (**d**) Bar graph showing that the expression of *Ccnd1* was not regulated by IL-15 in WT NK cells but was significantly altered in *Stat5* DKI NK cells. Expression is indicated by RPKM. (**e**) Bar graph showing that the expression of *Cdkn2b* was not regulated by IL-15 in WT NK cells but was significantly altered in *Stat5* DKI NK cells. Expression is indicated by RPKM. (**f**) Bar graph showing that the expression of *Cish* was potently induced by IL-15 in WT NK cells but not in *Stat5* DKI NK cells. Expression is indicated by RPKM.

Supplementary Figure 6

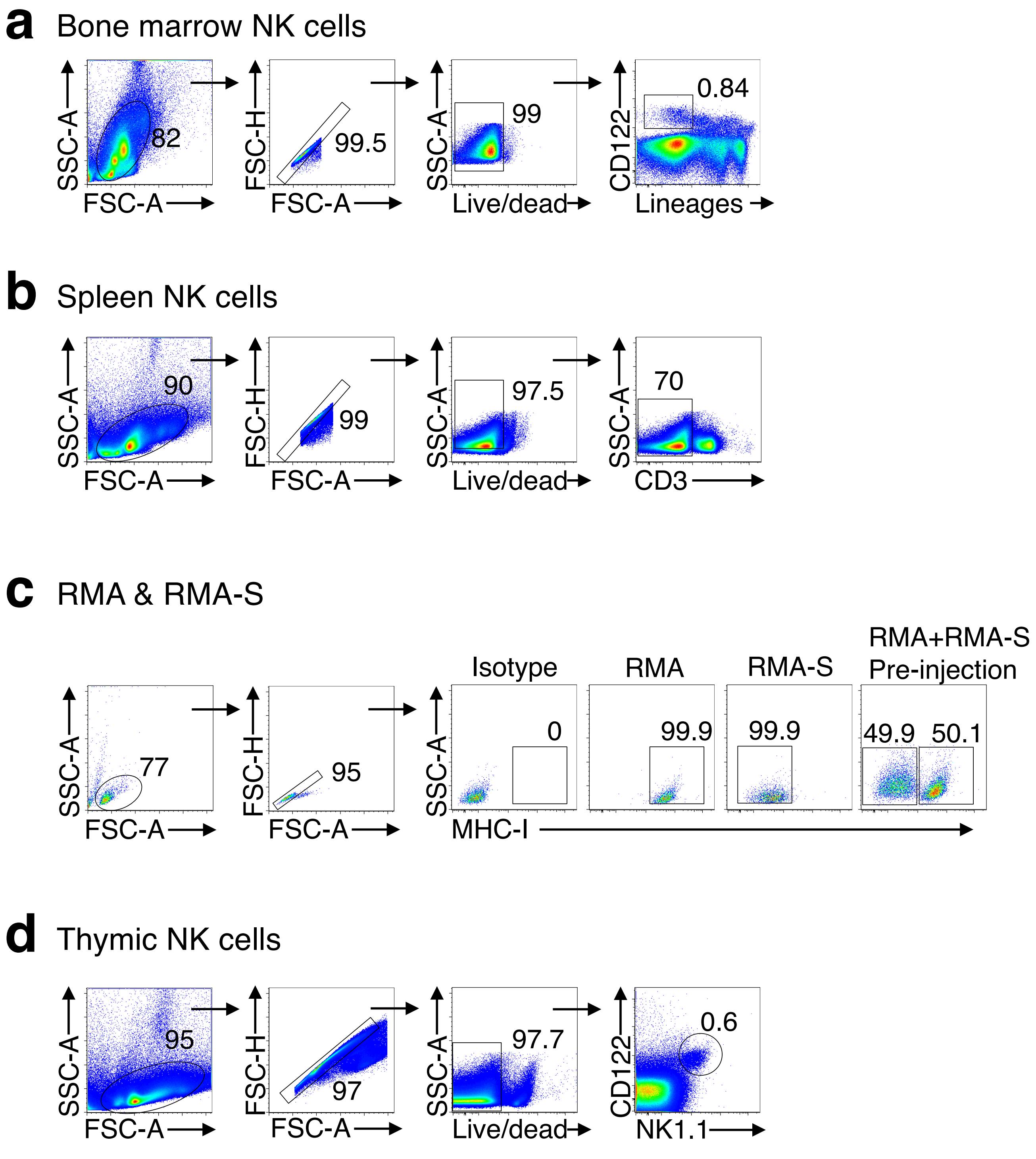

Supplementary Figure 6. Flow cytometry gating and sorting strategies. **(a)** Gating strategies for flow cytometric analysis of all bone marrow NK cells (Fig. 1b, 1d and 5c). **(b)** Gating strategies for flow cytometric analysis of all spleen NK cells (Fig. 1g, 5a, 5d, 6d, and Supplementary Fig. 2 and 5). (**c**) Gating strategy for Fig. 3c. (**d**) Gating strategies for Supplementary Fig. 1.
