# Supplementary material for: Critical functions for STAT5 tetramers in the maturation and survival of natural killer cells
Source: Nat Commun. 2017 Nov 6;8:1320. doi: 10.1038/s41467-017-01477-5 (PMC5673064; doi:10.1038/s41467-017-01477-5)
Supplement: Supplementary file 2 — Description of Additional Supplementary Files [file 41467_2017_1477_MOESM2_ESM.pdf]

Description of Additional Supplementary Files

File Name: Supplementary Data 1
Description: (**1a**) Shows the list of 892 genes that are differentially expressed in WT splenic NK cells during maturation. Their expression levels are shown in the indicated populations of WT and *Stat5* DKI NK cells. (**1b**) 462 genes are differentially expressed in WT NK cells during the $CD11b^{-}CD27^{+}$ to $CD11b^{+}CD27^{+}$ transition. Their expression levels are shown in the indicated populations of WT and *Stat5* DKI NK cells. (**1c**) 678 genes are differentially expressed in WT NK cells during the $CD11b^{+}CD27^{+}$ to $CD11b^{+}CD27^{low}$ transition. Their expression levels are shown in the indicated populations of WT and *Stat5* DKI NK cells. (**1d**) 357 genes are differentially expressed between WT NK Stat5 DKI $CD11b^{+}CD27^{low}$ NK cells. Their expression levels are shown in the indicated populations of WT and *Stat5* DKI NK cells. (**1e**) List of 7470 genes whose expression levels are not significantly changed (FC < 1.5) in WT NK cells during maturation. Their expression levels are shown in the indicated populations of WT and *Stat5* DKI NK cells. (**1f**) List of 82 genes encoding transcription factors whose expression is altered (FC > 1.5) in *Stat5* DKI NK cells. Their expression levels are shown in the indicated populations of WT and *Stat5* DKI NK cells. (**1g**) List of 77 genes encoding cytokines, chemokines, their receptors, and NK receptors whose expression is altered (FC > 1.5) in *Stat5* DKI NK cells. Their expression levels are shown in the indicated populations of WT and *Stat5* DKI NK cells.

File Name: Supplementary Data 2
Description: (**2a**) IL-15-regulated genes in WT and *Stat5* DKI NK cells. (**2b**) Genes whose expression levels are significantly altered in Stat5 DKI NK cells in response to IL-15 stimulation. (**2c**) Genes whose expression levels are not significantly changed in WT and *Stat5* DKI NK cells in response to IL-15 stimulation.

File Name: Supplementary Data 3
Description: (**3a**) List of genes that contain STAT5 tetramer binding sites in NK cells. (**3b**) STAT5 tetramer regulated genes in NK cells.
